# Extracellular RNA: A new perspective on the human pre-implantation embryos

**DOI:** 10.1016/j.xgen.2023.100472

**Published:** 2024-01-10

**Authors:** Jiye Fu, Jing Tu

**Affiliations:** 1 State Key Laboratory of Digital Medical Engineering, School of Biological Science and Medical Engineering, Southeast University, Nanjing 210096, China

## Abstract

It is currently a challenge to perform noninvasive molecular biological analysis of *in vitro* fertilized embryos. In this issue of *Cell Genomics*, Wu et al.^1^ developed a non-invasive method to evaluate human pre-implantation embryos by characterizing the extracellular RNAs in spent media from the culture of *in vitro* fertilization embryos.

## Teaser

It is currently a challenge to perform noninvasive molecular biological analysis of *in vitro* fertilized embryos. In this issue of *Cell Genomics*, Wu et al. developed a non-invasive method to evaluate human pre-implantation embryos by characterizing the extracellular RNAs in spent media from the culture of *in vitro* fertilization embryos.

## Main text

*In vitro* fertilization (IVF) requires embryo quality assessment. Currently, this is predominantly done with morphological assessment, which is a non-invasive tool for evaluating embryo quality. However, the human embryo has a limited number of physiology-associated and prognostic morphological features.^2^ Human embryonic transcription is initiated at the one-cell stage, with human embryos expressing up to 12,000 genes at each stage of development.^3^ Pre-implantation embryos are regulated to varying degrees by different genes at different stages of development, indicating that expressed genes may provide a means by which to assess embryo quality. However, the promise of this application has been limited due to the need for non-invasive sampling, preventing its adoption as a clinical method.

Trophectoderm biopsy is a widely used genetic test in clinical IVF, usually for the identification of *de novo* aneuploidies in embryos.^4^ Although implantation rates after embryo transfer are high after conducting a trophectoderm biopsy, debate continues over the accuracy (heterogeneity between the trophectodermal cell and the inner cell mass) and safety (due to the invasive sampling of embryos) of this method.^5^ Non-invasive access to the information present in the transcriptome of an embryo will assist in assessing the developmental status of the embryo and advance our understanding of embryonic development. In this issue of *Cell Genomics*, Wu et al.^1^ provide a viable path for a non-invasive means by which to sample embryos for IVF transfer.

**Figure 1:**
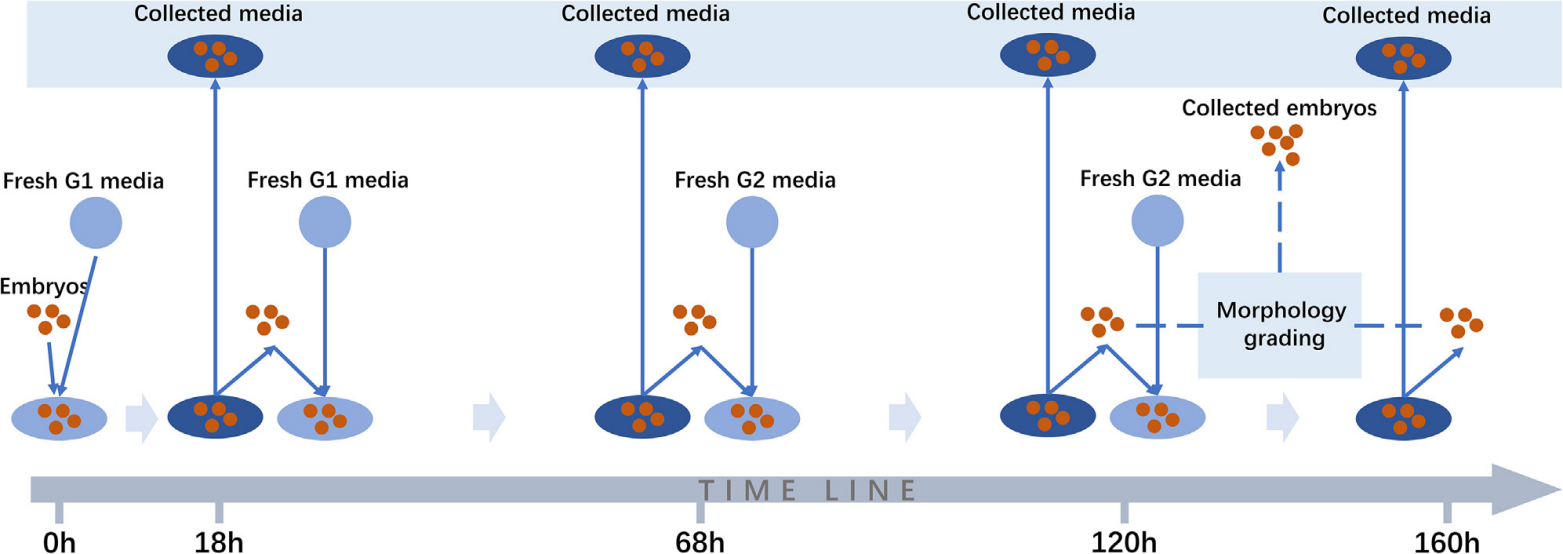
Illustration of the clinical procedure for human *in vitro* fertilization and spent media collection

This is not the first time embryo-relevant information has been extracted from the spent media of embryos or blastocysts. As early as 2016, Xu et al. extracted cell-free DNA from spent media as an indicator to investigate fetal aneuploidy,^6^ and Capalbo et al. extracted microRNA from spent media to assess human embryo reproductive competence.^7^ This year, Shi et al. extracted and characterized extracellular RNA (exRNA) from spent media and predicted the quality of blastocysts.^8^ However, Zhong and colleagues used the small input liquid volume extracellular RNA sequencing (SILVER-seq) technology they developed, which extracts exRNA from the spent media across the embryo culturing process, as shown in Figure 1.^9^ Using this approach, Wu et al. were able to characterize the extracellular transcriptome at five different stages of embryonic development, providing a wealth of extracellular transcriptome information associated with the embryonic development cycle. Their data identified approximately 4,000 exRNAs at each developmental stage, in which more than half are transcribed from coding genes. 245 high-quality libraries were established and used to generate a profile of the temporal extracellular transcriptome of human pre-implantation development.

Because exRNA reflects expression within the cell that is released to the extracellular space, the exRNAs obtained from spent media were only a subset of the cellular transcriptome. Wu et al. evaluated the correspondence of intracellular and extracellular RNAs of human embryos to evaluate the density and quality of information contained in this subset. A comparison of the degree of overlap of the expressed genes between embryo surveys and their exRNA samples demonstrated that the observed overlap was similar to that of single-cell RNA sequencing (scRNA-seq) studies of two independent embryos. Most SILVER-seq-detected exRNA genes overlap with scRNA-seq-detected genes from the corresponding stage, confirming that most exRNAs in the spent media correspond to the intracellular RNAs in human embryos. Characteristic time trajectories of exRNA during human pre-implantation development were further examined across different stages. Wu et al. then studied whether the exRNA in the culture media correlates with developmental arrest. Specific exRNAs that exhibited higher levels in the media of an arresting embryo compared to a developing embryo were designated arrest-correlated exRNAs. Arrest-correlated exRNAs were part of or responding to BMP signaling and negative regulators of the cell cycle.

Factors such as fertility willingness, demographic structure, and societal norms have resulted in IVF-assisted reproductive technology accounting for an increasing proportion of newborns.^10^ This increases the need for pre-implantation screening of embryos. After verifying that embryos have specific extracellular transcription profiles over developmental stages and that these profiles can be used to survey genes related to embryonic development, Wu et al. performed morphological assessment of every embryo at 120 and 160 h to generate a numeric value of which the mean of these values represents the average quality of the co-cultured embryos in a droplet. By training a random forest model using the exRNA levels of all the detected genes as inputs and the average quality value as the outcome, they identified genes with higher weights that were enriched with Gene Ontology (GO) terms corresponding to the Regulation of GTPase Activity and Spindle. Both of these GO terms are related to the regulation of asymmetric cell division. Because of this correlation, Wu et al. developed a simple binary classification model to assist in assessing the state of embryonic development, indicating that a machine-learning model utilizing exRNA profiles can accurately predict morphology-based embryo quality. This advance provides a new perspective for non-invasive assessment of embryonic development status, providing researchers with the opportunity to design more efficient and refined classification models.

It is worth mentioning that Wu et al. strictly followed current clinical standards and policy for IVF throughout the experimental process, which also led to some “defects” in the samples. Because, in clinical practice, co-cultured embryos show a better state of development, the acquisition of extracellular transcriptomes with individual embryos accounted for only a small part in this study, which inevitably led to an inability to explore questions such as the genotype and ploidy of each embryo. But this limitation comes from the need to adhere to scientific ethics. As more researchers perform similar studies increasing sample sizes, we anticipate the development of a more complete temporal extracellular transcriptome atlas to reveal the mechanism of pre-implantation embryo development.
